# Clustered regularly interspaced short palindromic repeats as an advanced treatment for Parkinson's disease

**DOI:** 10.1002/brb3.2280

**Published:** 2021-07-21

**Authors:** Arshad Mehmood, Wajid Ali, Zaheer Ud Din, Shuang Song, Muhammad Sohail, Wahid Shah, Jiangyuan Guo, Ruo‐Yi Guo, Ikram Ilahi, Suleman Shah, Fadhl Al‐Shaebi, Liaqat Zeb, Ernest Amponsah Asiamah, Zaid Al‐Dhamin, Hazrat Bilal, Bin Li

**Affiliations:** ^1^ Department of Neurology The Second Hospital of Hebei Medical University Shijiazhuang Hebei 050000 P. R. China; ^2^ Key Laboratory of Neurology of Hebei Province Shijiazhuang Hebei 050000 P. R. China; ^3^ Key Laboratory of Functional Inorganic Materials Chemistry, School of Chemistry and Materials Science Heilongjiang University Harbin 150080 China; ^4^ Institute of Cancer Stem Cell Dalian Medical University Dalian Liaoning 116044 China; ^5^ Institute for Advanced Study Shenzhen University Shenzhen 518060 China; ^6^ Department of Physiology Hebei Medical University Shijiazhuang Hebei 050017 China; ^7^ Department of Zoology University of Malakand Chakdara Khyber Pakhtunkhwa 18800 Pakistan; ^8^ Department of Genetics Hebei Medical University, Hebei Key Lab of Laboratory Animal Shijiazhuang Hebei 050017 China; ^9^ Department of Immunology, Key Laboratory of Immune Mechanism and Intervention on Serious Disease in Hebei Province Hebei Medical University Shijiazhuang 050017 China; ^10^ School of Bioengineering Dalian University of Technology Dalian Liaoning 116024 P. R. China; ^11^ Hebei Research Center for Stem Cell Medical Translational Engineering Shijiazhuang Hebei 050017 China; ^12^ Department of Traditional and Western Medical Hepatology Third Hospital of Hebei Medical University Shijiazhuang Hebei 050051 China; ^13^ State Key Laboratory for Chemistry and Molecular Engineering of Medicinal Resources, School of Chemistry and Pharmacy Guangxi Normal University Guilin Guangxi 541004 China

**Keywords:** clustered regularly interspaced short palindromic repeats‐associated protein 9, gene editing, induced pluripotent stem cells, neuroinflammation, Parkinson's disease

## Abstract

Recently, genome‐editing technology like clustered regularly interspaced short palindromic repeats (CRISPR)/Cas9 has improved the translational gap in the treatments mediated through gene therapy. The advantages of the CRISPR system, such as, work in the living cells and tissues, candidate this technique for the employing in experiments and the therapy of central nervous system diseases. Parkinson's disease (PD) is a widespread, disabling, neurodegenerative disease induced by dopaminergic neuron loss and linked to progressive motor impairment. Pathophysiological basis knowledge of PD has modified the PD classification model and expresses in the sporadic and familial types. Analyses of the earliest genetic linkage have shown in PD the inclusion of synuclein alpha (SNCA) genomic duplication and SNCA mutations in the familial types of PD pathogenesis. This review analyzes the structure, development, and function in genome editing regulated through the CRISPR/Cas9. Also, it explains the genes associated with PD pathogenesis and the appropriate modifications to favor PD. This study follows the direction by understanding the PD linking analyses in which the CRISPR technique is applied. Finally, this study explains the limitations and future trends of CRISPR service in relation to the genome‐editing process in PD patients' induced pluripotent stem cells.

## Graphical abstract

Parkinson's disease is a widespread, disabling, neurodegenerative disease induced by dopaminergic neuron loss and is linked to progressive motor impairment. Recently, genome‐editing technology like clustered regularly interspaced short palindromic repeats (CRISPR)/Cas9 has improved the translational gap in the treatments mediated through gene therapy. The advantages of the CRISPR system, such as, work in the living cells and tissues.

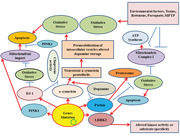

## 1 INTRODUCTION

Genetic screening research introduces the latest engineering named clustered regularly interspaced short palindromic repeats (CRISPR) with CRISPR‐associated (Cas). CRISPR/Cas9 genome editing technology has already been used to research and therapy various disorders such as various monogenic diseases, cancer, central nervous system (CNS) diseases, and AIDS (Knott & Doudna, 2018). By the genome‐editing tool kit, CRISPR offers the opportunity of modification, addition, or deletion of the genome in living cells. This technique comprises two main parts: Cas9, an endonuclease that causes double‐strand break (DSB) within DNA at a particular position, and a tiny guidance RNA that directs Cas9 to the purpose of interest, ensure genome editing accuracy and selectivity (Safari et al., 2019).

PD is a familiar and consistent neurodegenerative disease described through the destruction of dopaminergic (DAergic) nerve cells in substantia nigra (SN) (Emamalizadeh et al., 2014; Taghavi et al., 2018). The incidence of this neurological disorder is 1–2% in the populations aged above 65 years and may be found in individuals and all races across all over the world (Jamshidi et al., 2014; Schrag, 2018). In some cases, PD leads to a clinical motor disease named degenerative Parkinsonism, identified through rest tremor, bradykinesia, muscle stiffness, and postural dysfunction. PD neuropathology is described through DAergic neuron destruction, including the emergence of accumulated α‐synuclein involving Lewy bodies (LB). Therefore, an exception differs from the pathological morphology of classic PD. The long history of research suggests that gene mutations are capable of familial variants while the etiology of sporadic variants still unclear (Lubbe & Morris, 2014). The actual etiology of sporadic types still persists unclear though this is claimed whether neuroinflammation and oxidative stress can play a key role in the processes of sporadic PD (Nasrolahi et al., 2019).

**FIGURE 1:**
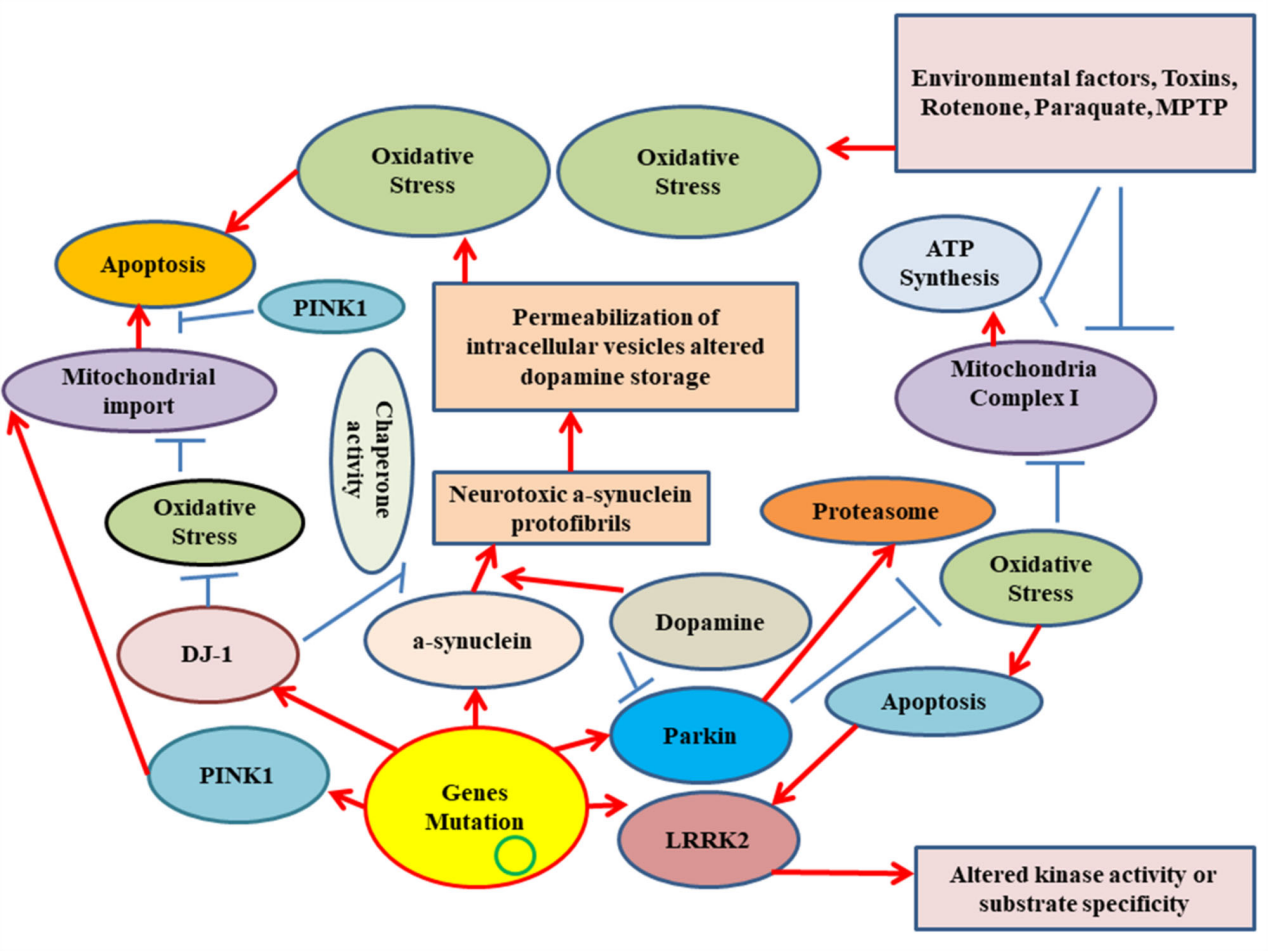
Representation of the vital molecular processes engages in the pathogenesis of Parkinson's disease (PD). Mitochondrial disruption and proteasomal abnormality play key roles that lead to the pathogenic mechanism. PD‐linked genes play key roles in protein folding, intracellular clearance processes, and modulation of mitochondrial homeostasis. The PTEN‐induced putative kinase‐1 (PINK1)‐parkin axis has active roles in the modulation of basic dynamic activities of mitochondria, including mitochondrial arrest, mitophagy, and fission‐fusion events. Parkin and PINK1 both independently regulate calcium homeostasis and mitochondrial biogenesis. Parkin and PINK1 mutations interrupt autophagy, which leads to neuronal cell apoptosis. The mutation of leucine repeat kinase‐2 and α‐SYN also affects mitochondrial activity by affecting oxidative phosphorylation, as well as, membrane potential, inducing the production of reactive oxygen species and releasing cytochrome‐c. Furthermore, DaisukeJunko‐1, Parkin, and PINK1 specifically stimulate the destruction of the proteasomal substrates, while α‐SYN can affect the function of the ubiquitin‐proteasome system. Subsequently, UCHL‐1 and synuclein alpha mutations disrupt α‐SYN destruction via autophagy‐mediated by a chaperone, results in α‐SYN accumulation and production of inclusion. ATP13A2 counterbalances the generation of α‐SYN accumulates. Some misfolded proteins and Alpha‐SYN aggregate in Lewy bodies, which are the pathological characteristics of the disorder

Mutations regulate autosomal‐dominant PD in synuclein alpha (SNCA), as well as, leucine repeat kinase‐2 (LRRK2), while the mutation causes autosomal‐recessive PD in Parkin, PTEN‐induced putative kinase‐1 (PINK1), and DaisukeJunko‐1 (DJ‐1), are depicted in (Table 1) (Scott et al., 2017). Some genes concerned with the pathogenesis of PD involved mutations in PLA2G634, SYNJ1, DNAJC1, ATP13A2, and FBX07. Genes such as CHCHD2, VPS35, and eiF4G1 are related to sporadic types of PD with different levels of penetrance. Findings from genome‐wide association studies revealed a link of at least 41 risk loci with PD pathogenesis (Chang et al., 2017). These findings suggested that SNCA is one of the main strong risk loci linked to PD sporadic type (Devine et al., 2011). Moreover, all genes inspected in monogenic PD forms cannot completely show the etiology of all PD. However, the processes of gene mutations linked to the PD pathogenesis are shown in (Figure 1).

**TABLE 1:** Genes engaged in the pathogenesis of Parkinson's disease, neuropathology, function, and phenotype

Genes	Genes function	Phenotype	Results of gene mutation
D‐J1	Most organs and tissues, including the brain have DJ‐1 protein. This protein suggests having secured cells against oxidative stress and plays a role as a chaperone molecule. DJ‐1 facilitates the assembling of new proteins into the appropriate three‐dimensional form and refolding the degraded ones	Early‐onset PD	LB pathology
PINK1	The PINK1 gene generates a protein known as PTEN‐induced putative kinase 1. This protein acts within mitochondria, securing mitochondria against the damaging impact of cellular oxidative stress	Early‐onset PD	The presence of LB in reticular nuclei of the brainstem and loss of the neuron in the SN pars compacta
SNCA	The α‐synuclein protein is a component of the SNCA gene that spreads widely in nerve cells; its role is not yet completely known however can play a role in regulating dopamine neurotransmission and vesicular neurotransmission	Early‐onset PD	Extensive involvement of LB in the brain and cerebral cortex, neural degeneration in SN, as well as, LC
RAB39B	The RAB proteins like RAB39B are linked to the family of GTPases. Vesicles trafficking in the compartments of the membrane are regulated by RAB39B	X‐linked early‐onset PD	Widespread loss of dopaminergic neuron in SN and disorder of classical LB
PARKIN	Parkin is also an E3 ubiquitin ligase and plays a role in the proteasome‐dependent deterioration of protein. It damages misfolded and excessive proteins along with ubiquitin	Early‐onset PD	Lack of LB, apoptosis of the dopaminergic neuron from the SN, and neurofibrillary tangle pathology in the cerebral cortex as well as brainstem
LRRK2	The component of the gene LRRK2 belongs to the protein Roco family. It plays roles in vesicular transport, autophagy, and cytoskeletal dynamics	Classical PD	Heterogeneous: the destruction of neurons in the SN and involvement of LB in the brain and; certain cases: Lack of LB and neural nigral deterioration, neurofibrillary tangle pathology

Abbreviations: PD, Parkinson's disease; SN, substantia nigra; LB, Lewy body; LC, locus coeruleus.

Also, it is suggested that neuroinflammation is a primary cause of the onset and development of sporadic PD (Ghosh et al., 2016). In PD, the hyperactivation of microglia or astrocytes is caused by various chemical or physical insults. Neurotoxic substances of the environment such as, Mn (Luo et al., 2019), 1‐methyl‐4‐phenyl‐1,2,3,6‐tetrahydropyridine (MPTP) (Ghosh et al., 2016), and several pesticides induce neuroinflammation by a disturbance between a glial cell and neurons signaling mechanisms (Song et al., 2019). Stimulation of microglia enhances the release of pro‐inflammatory cytokines. Gliosis leads to the nuclear translocation of NF‐κB, stimulation, and activation of the pro‐inflammatory cytokines such as interleukin (IL)−1beta, IL‐6, tumor necrosis factor‐α (TNF‐α), cyclooxygenase 2, prostaglandins E2, nitric oxide, and inducible nitric oxide synthase (Luo et al., 2019). The long history of research showed that under different conditions, though neuroinflammation and mitochondrial abnormalities interact together in the pathogenesis of PD (Sarkar et al., 2017).

Currently, a recent approach of CRISPR‐related PD studies is growing and could potentially trigger tectonic alters in treating PD and other neurologic disorders.

Recently, a huge flow of CRISPR‐related PD research is developing, and it is important that researchers of the PD must gain knowledge regarding how this powerful technique plays a role. By using powerful possibilities such as epigenetic alteration, transcriptional activation/repression, gene knocking in/out, and gene alteration, the CRISPR/Cas9 technology leads to the direction for PD therapy and improves genomic research for the pathogenesis of PD.

This review analyzes the structure and role of the CRISPR system in order to overcome the vital advancement in identifying the genetic pathogenesis of PD regulated through the CRISPR technique. Continuously, the genetic factors associated with the pathogenesis of PD are explained. Furthermore, we also review researches that applied CRISPR to examine PD and the models of PD created through the genome‐editing technique. Finally, the limitations and future trends of induced pluripotent stem cells (iPSCs) and the genome editing creation regulated through the CRISPR technique are described.

## 2 PATHOPHYSIOLOGY OF PARKINSON'S DISEASE

Parkinson's disease is represented by DAergic neuron destruction in the SN. The pathological characteristic of PD is the Lewy body, a neural inclusion comprising widely of α‐synuclein protein accumulations. The Braak postulate is the most frequently cited model for describing the neuropathological development of PD. This model indicates that PD (stages 1 and 2) starts in the medulla and olfactory bulb. This earlier pathology is linked with symptoms that arise similar to the onset of movement syndromes, such as, rapid eye movement sleep behavior disorder (organisms lose regular rapid eye movement sleep paralysis and actually behave on their dreams during sleeping), and decreased smell. Furthermore, in stage‐3 and stage‐4, the disease advances to the midbrain, basal forebrain, and SN pars compacta. Pathology in these regions is linked to classic PD motor symptoms. At this stage, PD is normally diagnosed. Moreover, in developed PD, the pathology advances towards cerebral cortices with cognitive‐onset hallucinations and impairment (Braak et al., 2003).

## 3 CLINICAL PRESENTATION OF PARKINSON'S DISEASE

PD triggers motor and nonmotor symptoms (Table 2). Motor symptoms are physical and movement activities: Slowness, tremor, imbalance, and stiffness. Nonmotor (nonmovement) symptoms affect multiple organs, such as, genitourinary and gastrointestinal systems, and are heterogeneous. Patients cannot responsibly be volunteering nonmotor symptoms even though they feel embarrassed, while the time of appointment is concentrated on motor symptoms, and patients are unsure whether the symptoms could be PD‐associated (Chaudhuri et al., 2010).

**TABLE 2:** Motor and nonmotor symptoms of Parkinson's disease

Symptoms	Key elements
**Motor**	
Rest tremor^a^	A 4‐ to 6‐Hz tremor in such a completely rest limb, which temporarily disappears when the limb is held return and then outstretched (reemergent tremor) and is not present in the movement
Rigidity^a^	Involuntary, velocity‐independent resistance to a joint (e.g., wrist, elbow) passive movement through an examiner, without or with a cogwheel phenomenon
Postural instability	Impairment of balance affects the ability of a person to maintain or change postures such as standing or walking; normally a feature of late Parkinson's disease
Bradykinesia^a^	Progressively small movements and slowness (hypokinesia) as a person repeats an action several times in a line (e.g., tapping thumb and index finger, closing and opening fist)
**Nonmotor**	
Psychiatric disturbances	Psychosis, anxiety, apathy, depression
Cognitive impairment	Dementia or mild cognitive impairment, frequently initially influencing attention, visuospatial activities, and executive
Sleep dysfunction	Signs of rapid eye movement sleep behavior disorder, sleepiness during daytime, sleep maintenance insomnia
Autonomic dysfunction	Urinary frequency and urgency, constipation, delayed gastric emptying, erection problems, orthostatic hypotension, the variability of blood pressure
Olfactory loss	Hyposmia, absent or decreased sense of smell
Other	Hypophonia (voice softening), fatigue, sialorrhea, trouble during swallowing

^a^ Primary feature indication.

Individuals diagnosed with PD normally produce nonmotor symptoms for years before movement symptoms begin, but commonly they do not discuss these symptoms unless directly asked. These nonmotor prodromal characteristics involved rapid eye movement, sleep behavior disorder, smell loss, constipation, dysfunction of urine, orthostatic low blood pressure, depression, and prolonged sleepiness during the daytime. These symptoms are not related to PD, while when they coexist, the risk of upcoming PD diagnosis is higher. Rapid eye movement sleep behavior disorder is significantly connected with an enhanced risk of subsequent examination of PD, especially if detected on polysomnography (Berg et al., 2015). Over 90% of people with idiopathic rapid eye movement sleep behavior disorder subsequently generate a synuclein‐associated neurological disorder, typically PD or an associated disease (LB, dementia, and multiple system atrophy) (Galbiati et al., 2019). An approximated 30% to 50% of Parkinson's patients have rapid eye movement sleep behavior disorder (Howell & Schenck, 2015).

Prodromal signs and symptoms are related to early PD pathology of the brainstem. When neuropathological development results in loss of nearly half of the cells in caudal SN, motor symptoms and signs of PD appear, and people present with family or personal concerns about the progressive onset of slowness, generalized stiffness (not joint‐specific), and rest tremors (Fearnley & Lees, 1991). On the other hand, around 20% of people with PD have no resting tremors (Hughes et al., 1993; Pasquini et al., 2018).

## 4 CLUSTERED REGULARLY INTERSPACED SHORT PALINDROMIC REPEATS/Cas9 SYSTEM: TECHNOLOGY FOR GENOME‐EDITING

Traditionally, small interfering RNA and short hairpin RNA (shRNA) have been applied in genome editing (Safari et al., 2017; Safari et al., 2016). Furthermore, the most innovative characteristic of the past century was the innovation of a new powerful technology of gene‐editing called CRISPR. The research explosion in 2013 touched on this gene‐editing technology, fascinating the global scientific society. It might not be a stretch to suggest that it can represent a breakthrough in our age. The CRISPR/Cas9 technique could be applied to cut and edit every genome anywhere in any interesting position. In the intended DNA sequencing directed through sgRNA, the nuclease function of the Cas9 protein triggers DSB while it assesses the specificity and precision of the CRISPR technique. In the repair of DSB, the repair system of cells taking action and fixes this site by employing 2‐techniques, such as, non‐homologous end joining and homology‐directed repair. These 2‐techniques permit direct homologous recombination or random mutation like insertion/deletion (in/del) in the availability of the donner template. However, the deactivation of Cas9 (dCas9) nuclease provides a useful technique for the alteration of gene transcription. Inclusion of the activator domains such as VP64 and V16 results in stimulation of gene of interest. By comparison, the dCas9 fusion and with the repressor domain, such as, the KRAB, permit intended gene repression. In the art state, epigenetic alteration is induced by joining the DNA demethylase TET or DNA methyltransferase 3A (DNMT3A) or p300 core with dCas9 (Safari et al., 2019).

However, CRISPR is always a powerful gene tool even though it also has a certain drawback, such as, the off‐target effect that makes doubtful the application of this latest technology in treatment strategies. Furthermore, to enhance its specificity, researchers have discovered an engineered model of Cas9 such as, nickase Cas9 (Cas9n), high fidelity Cas9, and enhanced specificity Cas9 (Ran et al., 2013). As the high‐throughput CRISPR technique, Cas9n comprises two mutated nucleases of Cas9 that trigger the induction of endonuclease enzymes of 2 FOK1, two sgRNAs, and single‐strand nicks. In the intended gene, this complex triggers DSB even with higher specificity, but it requires two sgRNAs to identify the target position to improve the accuracy of this CRISPR system (Safari et al., 2019). CRISPR has flexibility and usefulness, which delivers a promising possibility for applying this technique in diverse research areas, such as, functional gene annotation, modeling of animals, and treatment strategies.

## 5 GENETIC CAUSES IN PARKINSON'S DISEASE PATHOGENESIS AND PREVENTION

### 5.1 Synuclein alpha

In 1997 Polymeropoulos was the first to discover the missense mutation in the SNCA gene. The discovering of this mutation, and the detection of risk factors of familial type, confirmed a clear association between the familial and sporadic types of PD (Polymeropoulos et al., 1996). It is reported, as a typical pathological characteristic of PD, LB includes α‐synuclein protein. Therefore, 5 different types of mutations in SNCA have been confirmed, such as, two‐point mutations and three‐missense mutations. These different mutations result in different ages of onset and various diagnostic presentations. Missense mutations (E46K, A30P, and A53T) cause extremely advanced Parkinsonism early‐onset and with a proper initial reaction to L‐dopa. Furthermore, point mutations like G51D and H50Q induce different clinical symptoms with various onset and severity of the disorder (Ferreira & Massano, 2017).

The etiological variables for PD are tandem repeats within the SNCA locus, such as triplications and duplications. Subsequently, findings have demonstrated that two extreme copies of the genomic region comprising the gene of α‐synuclein result in the PD familial type. Moreover, the quantity of SNCA copies assesses the PD severity. Research of duplicated SNCA has shown that 50% improvement in the expression of SNCA leads to the generation of an autosomal‐dominant PD type, while this specifies that risk variants linked with PD could induce an improved SNCA expression (Kim et al., 2012).

Neuropathological studies of SNCA‐associated PD demonstrated severe neural deterioration in the locus coeruleus (LC) and SN region. Moreover, in the patient with the SNCA triplication, the widespread of LB is found in the cerebral cortex and brain stem (Farrer et al., 2004).

### 5.2 Leucine Repeat Kinase‐2

In LRRK2, the autosomal‐dominant mutation was detected as the fundamental genetic cause of PD. Also, LRRK2 is a sufficient multidomain protein in the ROC‐COR (ROCO) superfamily (Marín, 2008). Furthermore, the accurate therapeutic role of this protein is still unclear, while its inclusion in the outgrowth of a neurite, protein autophagy, cytoskeletal maintenance, vesicle trafficking, and the immune system is highly developed (Anand & Braithwaite, 2009).

It is already assessed that even 4% of familial types of PD carry various forms of LRRK2 mutation (Hasegawa et al., 2009). In the etiology of PD, a high proportion of mutations have already been estimated to play a role. G2019S is a very common mutation of LRRK2 in familial (≈3–6%) and sporadic (1%) types of PD.

In sporadic conditions, the clinical presentation of LRRK2‐associated Parkinsonism is not different. The age for the onset of this form of PD is about 60 years. Noteworthy, the tremor during dystonia and presentation is extremely frequent in G2019S patients, especially compared to non‐mutated patients of LRRK2. It was confirmed that abduction‐adduction tremors in the lower limbs might be observed as a diagnostic marker. Moreover, a comparison of dyskinesia as an investigating level of PD frequency in LRRK2‐associated Parkinsonism and idiopathic PD showed a less frequent format of LRRK2‐linked Parkinsonism (Healy et al., 2008).

Some pathological investigations in the same family were confirmed heterogeneous neuropathological results of LRRK2‐linked PD. Furthermore, most of the LRRK2 instances reports show the neurons' destruction in SN and the existence of LB within the brainstem. A few cases indicate glial cytoplasmic involvements reminiscent of multiple system atrophy, neural nigral destruction without the LB, and neurofibrillary tangle pathology (Zimprich et al., 2004).

### 5.3 Parkin

Mutations in the gene Parkin (PRKN; it is also called PARK2) are the extremely frequent cause of early‐onset autosomal recessive Parkinsonism. The PRKN encoded Parkin E3 ubiquitin ligase that is involved in the quality regulation of mitochondria and turnover. Predominantly, Parkin is the cytosolic protein while it is confined to the mitochondria under certain situations (Sarkar et al., 2017). PINK1 modulates the function of Parkin by phosphorylation activities (Arkinson & Walden, 2018). PARKIN and PINK1 play crucial roles in the quality regulation of mitochondria and mitophagy (Lazarou et al., 2015).

Cytosolic PARKIN is triggered by the aggregation of PINK1 on the layer of abnormal mitochondria. The arranged ubiquitin chains on several proteins of mitochondrial surface are modified by stimulated S65‐phosphorylation and PARKIN regulated through PINK1 (Rose et al., 2016). On mitochondria, the aggregation of S65‐phosphorylated ubiquitin (pUb) starts the signaling process to activate the autophagy machinery also for the specific removal of abnormal mitochondria (Lazarou et al., 2015).

It has been confirmed that about 19% of early‐onset isolated cases of PD carry mutations in the PARKIN. Moreover, heterozygous PARKIN mutations were also described in certain familial of PDs. However, this evidence suggests that a single allele mutated PARKIN carriers could be at the risk of progressing PD. Furthermore, PARKIN mutations and their carriers have a therapeutic profile represented by a gradual advancement of disorder and a proper reaction to levodopa, with extreme early‐onset of dyskinesias (Lincoln et al., 2003; Lohmann et al., 2003). PARKIN and PINK1 mutations result in also the familial sub‐type, which suggests that damaged mitophagy is also a cardinal trait of PD (Kitada et al., 1998).

It is confirmed that PARKIN‐associated PD, with regards to neuropathology, the lack of LB, widespread destruction of SN DAergic neurons, the existence of neurofibrillary tangles in the brainstem and cerebral cortex (Mori et al., 1998). Moreover, recent studies show the existence of LB in LC and SN and α‐synucleinopathy localized to mesencephalic reticular formation (Pramstaller et al., 2005).

### 5.4 DaisukeJunko‐1

It was confirmed in 2003, in addition to DJ‐1 gene homozygous deletion, a missense mutation triggered the autosomal recessive early‐onset of PD (Annesi et al., 2005; Lohmann et al., 2003). Up to date, multiple novel DJ‐1 mutation has already been reported in early‐onset PD patients. Interestingly, these types of mutations are very rare and may be identified in just 1% of patients with early‐onset PD (Abou‐Sleiman et al., 2003). In addition, DJ‐1 protein exists in other organs, including the brain. Moreover, cellular response to oxidative stress triggers the transition of the cytosolic DJ‐1 protein to the mitochondria. Furthermore, the endogenous DJ‐1 has been obviously identified in the intermembrane space and mitochondrial matrix. This fact supports the concept that DJ‐1 can play a role in cellular defense against oxidative stress. Moreover, DJ‐1 serves as a chaperone molecule to facilitate appropriate three‐dimensional proteins and re‐fold the destroyed proteins (Zhu et al., 2017). DJ‐1 neuropathology is not completely understood; however, the current analyses have reported the pathological function of LB in DJ‐1‐related PD (Taipa et al., 2016).

### 5.5 PTEN‐induced putative kinase‐1

PINK1 mutations have been identified initially in a family of Sicilian with early onset of autosomal recessive Parkinsonism. Moreover, most PINK1 mutations are missense, while some copy quantities of mutations and exonic reinterpretations have been confirmed (Samaranch et al., 2010). In homozygous and compound heterozygous manifestations, these mutations were reported in sporadic and familial (2–4%) instances. The role of heterozygous mutations in the pathogenesis of PD promotes the hypothesis that carriers of the singly PINK1 heterozygous mutation are at the imminence of PD (Nuytemans et al., 2010). This may be claimed whether the clinical description of PINK1‐associated PD is on average with DJ‐1/Parkin‐associated PD instances. The low progressive disorder, which is very sensitive to levodopa treatment, can characterize the morphology of this PD subtype. In this case, the roles of Parkin and PINK1 are strongly interconnected. It may be reasonable to confirm that the Parkin/PINK1 axis regulates basic mitochondrial dynamic characteristics such as mitochondrial arrest, mitophagy, and fission‐fusion events. Also, Parkin and PINK1 can independently control calcium homeostasis and mitochondrial biogenesis. Furthermore, PINK1 regulates autophagy through interaction with protein Beclin‐1 (Jian et al., 2018).

Neuropathological analysis in a PINK1‐associated PD patient has been indicated the loss of neurons and LB aggregation in areas such as SN pars compacta, Meynert nucleus basalis, and brainstem nuclei (Valente et al., 2004).

### 5.6 RAB39B

Rab GTPase is an insufficient weight protein, which supports regulate vesicle trafficking (Cheng et al., 2002). RAB39B is among the three X‐linked RAB genes that are significant to the brain (Wilson et al., 2014). Up to date, many researchers reported that mutations in RAB39B lead to X‐linked intellectual disorder and PD. The deletion of a missense mutation, ∼45‐kb in RAB39B, RAB39B, and p.Trp186 stop mutation, reduces the function of RAB39B and improves the transfer to Parkinsonism (Lesage et al., 2015). In neuropathology, postmortem studies have shown extreme general DAergic neural destruction in SN and extensive classical LB disorder in RAB39B‐associated PD (Wilson et al., 2014).

### 5.7 P13

The inclusion of mitochondrial abnormalities has already been identified in patients with sporadic PD pathogenesis. Furthermore, several toxin‐induced Parkinsonism and patients of familial PD show mitochondrial abnormality. Therefore, suppressing the mitochondrial abnormalities can be a crucial technique to resist PD. As a mitochondrial matrix protein, p13 (c7 or f55) decreases in the time of pancreatic cells exposure towards oxidative stress (Higashi et al., 2015). Moreover, p13 uses its effects by linking to the sub‐units of the electron transport chain complexes I, as well as, V named ATPAF2 and NDUFAB1 (Floyd et al., 2016). In recent times, it has already been demonstrated that p13 specifically controls the function of complex I and facilitates the assembly of complex I by an increase in the expression of p13. This finding demonstrates the interconnection of p13 in the models of PD. Recent studies reports have shown that the decrease in the expression of p13 inhibits mitochondrial deformities identified in genetic and toxin‐induced models of PD. Moreover, the regulatory effects of p13 on mitochondrial activity have been confirmed in either in vivo or in vitro models of PD (Inoue et al., 2018).

### 5.8 Ghrelin

Ghrelin is a receptor‐activated hormone and G‐protein attached receptor, the growth hormone secretagogue receptor (GHS‐R). Therefore, this protein controls the secretion of growth hormone, the performance of memory, intake of food, and seeking behavioral reward (Abizaid et al., 2006). Ghrelin strongly regulates neuronal function, and GHS‐R expression has also been detected in various brain regions such as the ventral tegmental region, SNc, hippocampus, and hypothalamus (Osterstock et al., 2010). The electrical action of Ghrelin activates DAergic neurons in SNc and improves the dopamine level within the striatum (Shi et al., 2013). The neuroprotective effects of Ghrelin are also well known; as seen in the MPTP PD model, it prevents the destruction of DAergic neurons in SNc (Andrews et al., 2009).

### 5.9 Prokineticin 2

Prokineticin belongs to the family of AVIT protein and comprises prokineticin‐1 and prokineticin‐2 (PK2). By the PKR1 and PKR2 receptors of prokineticin, these two chemokines signal, these were known as prokineticin due to their potential of contracting smooth muscle of the gastrointestinal system from the isolated ileal parts (Pitteloud et al., 2007). PK2 modulates a wide range of mechanisms such as pain reception, neurogenesis, hematopoiesis, circadian rhythm, and reproductive functions. PK2 modulates neurogenesis in the brain by leading the relocation of progenitor cells from the subventricular region in the olfactory bulb biogenesis. It also controls circadian rhythms by playing a role as the output molecule also from the suprachiasmatic nucleus (Cheng et al., 2005). In recent times, the inclusion of PK2 in the expenditure of energy and thermoregulation by the hypothalamus has been confirmed 8. Findings of the latest research found that PK2 mRNA has been overexpressed by TNF‐a during DAergic cell death (Gordon et al., 2012). Furthermore, it is postulated that PK2 is the potential mediator of signaling secreted in DAergic deterioration.

### 5.10 Protein Kinase Cδ

Protein kinase Cδ (PKCδ) belonging to a novel isoform family of PKC, which is involved in various signal transduction mechanisms such as cell cycle development, proliferation, apoptosis, and differentiation. Findings demonstrated that Kinase expression is strong in DAergic cell lines primary, DAergic cultures, and SNpc DAergic neurons (Zhang et al., 2007). PKCδ is cleaved proteolytically through caspase‐3 in the DAergic cells in the time of neurotoxic stress, which triggers apoptotic death of neural cells (Hanrott et al., 2008). It is also confirmed that activation of TNFα receptors within DAergic neurons induces apoptosis and proteolytically stimulates PKCδ (Gordon et al., 2012). PKCδ controls immune and inflammatory reactions in the cells of peripheral immune such as B cells, neutrophils, and macrophages, together with the function in apoptosis. Studies deliver the fact that activated microglia stimulated through different inflammatory factors contains LPS, TNFα, and accumulated α‐synuclein leads to the stimulation of PKCδ. Furthermore, this stimulation is followed through the activity of kinase and PKCδ gene‐upregulation, which promotes the postulate that microglial stimulation is combined with an improved expression of PKCδ protein and related activity of kinase (Gordon et al., 2016).

Findings deliver proof that the activated microglia triggered through different inflammatory factors such as accumulated α‐synuclein, TNFα, and LPS leads to the stimulation of PKCδ. However, this stimulation is followed through the activity of kinase and upregulation of the PKCδ gene, which promotes the postulate that the microglial stimulation is combined with an improved expression of the PKCδ protein and associated activity of the kinase. Moreover, the application of pesticides to N27 DAergic neural cells such as endosulfan readily causes autophagy. Furthermore, the long‐term application of endosulfan stimulates apoptotic signals causes the stimulation of caspase‐2 caspase‐3 and proteolytic stimulation of PKCδ. Subsequently, the stimulations of this signal mechanism result in cell death. Moreover, this suggests that apoptosis accompanying autophagy in the neurotoxicity of endosulfan (Song et al., 2019).

Different reports have been investigated the interaction between PD pathogenesis and specified genes. The advancement of genetic engineering technology could make this field of researches further possible. The latest established CRISPR‐Cas9 technologies of genome editing have allowed efficient as well as accurate epigenetic and genetic modification of genomes. The CRISPR system has reshaped cellular function study in disease and health. PD as a neurodegenerative disorder, predicting affect multiple people within a decade ahead. Therefore, investigations are currently ongoing to better understand the fundamental reasons for PD and to discover appropriate clinical strategies.

## 6 MECHANISM OF GENE EDITING BY CLUSTERED REGULARLY INTERSPACED SHORT PALINDROMIC REPEATS/Cas9 IN PARKINSON'S DISEASE

### 6.1 Synuclein alpha gene editing by clustered regularly interspaced short palindromic repeats

SNCA is among the typical risk loci related to the patients of sporadic PD, based on genome‐wide association studies data (Schrag, 2018). It is important to note that none of the sporadic PD‐linked single‐nucleotide polymorphisms (SNPs) affects the a‐synuclein protein‐coding sequence (Lubbe & Morris, 2014). Furthermore, it has already been confirmed that these variants can improve the levels of gene expression (Scott et al., 2017); however, the generation of LB and accumulation of irregular protein are classical pathological characteristics of PD. Moreover, to confirm this postulate, Soldner et al. used the technology of genome‐wide association studies, iPSCs, and CRISPR/Cas9 techniques to identify various SNCA variations, including SNP to modify appropriately. Furthermore, these data were used to investigate the interaction between PD pathogenesis and SNCA variants. In this technique, a typical SNCA variant was applied which embedded in a distal enhancer component of non‐coding. It is postulated that this variant modulates SNCA expression by improving the specificity of transcription agents to this enhancer component.

To determine this postulate, researchers applied CRISPR/Cas9 to sequentially eliminate the enhancer area containing two risk‐related SNPs, rs3756054 and rs356168, in the embryonic stem cells of humans. Afterward, inserting one of these two enhancers deleted alleles, including one potential variant, results in the production of four heterozygous cells. Significantly, the deleted cell of the homozygous enhancer was applied as a control. Afterward, the cells differentiated into neurons or neural precursors. Furthermore, the TaqMan SNP genotyping assays were used to determine the equivalent levels in the transcriptions of SNCA in the genetically modified cells. However, results indicated that a typical risk‐related variant could modify the a‐syn expression by modifying the ability of transcription factors (TFs) to the enhancer. This is even the first study in which the researchers explained the effects of genetic variants on the risk of PD at the molecular side by employing CRISPR/Cas9 technology based on genome‐wide association studies data. Moreover, experimental data reflect attention on the inclusion of genetic variants of non‐coding in elevation of the expression levels of the gene. Furthermore, in this analysis, the productive use of genome‐wide association studies data, TaqMan qRT‐PCR, and CRISPR/Cas9 research promote the quantitation of allele‐specific levels of expression in one multiplex reaction while still there are some limitations (Soldner et al., 2016).

To this purpose, Chen et al. developed a pluripotent stem cell engineered with removed or reduced alleles of SNCA that encoded for the α‐syn. Applying the technology of Cas9n with four gRNAs, the first SNCA gene coding exon was properly removed in human embryonic stem cells to generate multiple SNCA‐/‐clonal cell lines and SNCA ±. Moreover, the iPSC line was manipulated that harbored three genes of α‐synuclein and produced triple knockout cell lines.

Effective differentiation of all these cell lines genotype to mDA neurons specified that knockout of the protein α‐synuclein did not affect the differentiation ability of pluripotent stem cells in this neural sub‐type. Effective experiments and findings of transcriptome showed that the numbers of DAergic neurons within substantia nigra Snca‐/‐mice were common, and the content of dopamine in the striatum was decreased (Abeliovich et al., 2000). Moreover, the findings of this analysis suggested that free grafts of α‐synuclein can have a specific defensive effect from MPTP neurotoxins.

Furthermore, in this background, the CRISPR technique can be shown as a functional technology to produce SNCA ± and SNCA‐/‐mDA neurons through human embryonic stem cells or iPSCs. Also, different modified variants of the Cas9 protein, such as Cas9n, can provide a new platform to produce the new generation of Parkinson resistance cells (Chen et al., 2019).

### 6.2 Parkin gene editing by clustered regularly interspaced short palindromic repeats

PARKIN and PINK1 are crucial in mitophagy and mitochondrial quality regulation. In earlier analyses, screens of RNAi were used to detect regulators of the PARKIN/PINK1 mediated mitophagy (Mccoy et al., 2014). Researchers have applied CRISPR/Cas9 gene‐editing engineering to explain on set mitophagy threshold of the cells as a screening technique that aims to overcome RNAi for phenotypic screening (Dejesus et al., 2016).

The phenotypic genome‐wide CRISPR/Cas9 pooled screen was conducted to identify PARKIN regulators. In this project, the researcher used cells that express the reporter protein of PARKIN through the endogenous PARK2 promoter. In this analysis, the level of PARKIN assessed the kinetics of the pUb aggregation. The findings from this project indicated that 53 negative and positive variables were engaged in the control of the PARKIN.

According to the results of bioinformatics, it is claimed that transcription repression negatively controls the endogenous level of PARKIN. Incredibly, in different cell forms, the decrease of THAP11 affects both levels of PARKIN protein and pUb aggregation. Furthermore, CRISPR/Cas9 targeting THAP11 in iPSC‐derived inducible Neurogenin‐2 neurons led to improved pUb aggregation and de‐repression of PARK2 transcription (Potting et al., 2018).

Although the accurate mechanism through which PARKIN regulates syndromes like PD still remains unspecified, scientists have developed a monogenic type of PD‐particular iPSCs derived dopamine neurons that harbor mutations of PARKIN. Therefore, CRISPR/Cas9 can create PARKIN knockout and knockin iPSCs through the insertion of donor series in exon‐2 of the gene silencing the function of the PARKIN. Analysis of this isogenic cell line showed a substantial decrease in the expression of mRNA of GHSR1a and GHSR1b. However, findings showed the effect of the PARK2 mutation even effect also on the expression of the GHSR1a. Furthermore, PARK2 mutation can also modify the shift of ubiquitin onto the substrate proteins while it could alter the transcription level of GHSR1a (Suda et al., 2018).

### 6.3 P13 gene editing by clustered regularly interspaced short palindromic repeats

It is already explained that p13 is engaged in the regulation of mitochondrial activity, while it is suggested as a potential treatment for PD. Inoue et al. researched the levels of P13 for both genetic and toxin‐induced PD models to assess this postulate. It is reported that levels of p13 are downregulated in vivo and in vitro toxin‐induced PD models (neuroblastoma cells of mice and humans). Even though closely related lower levels were not detected in PD, a variation in both chronic and acute models may be an underlying process of this finding.

**FIGURE 2:**
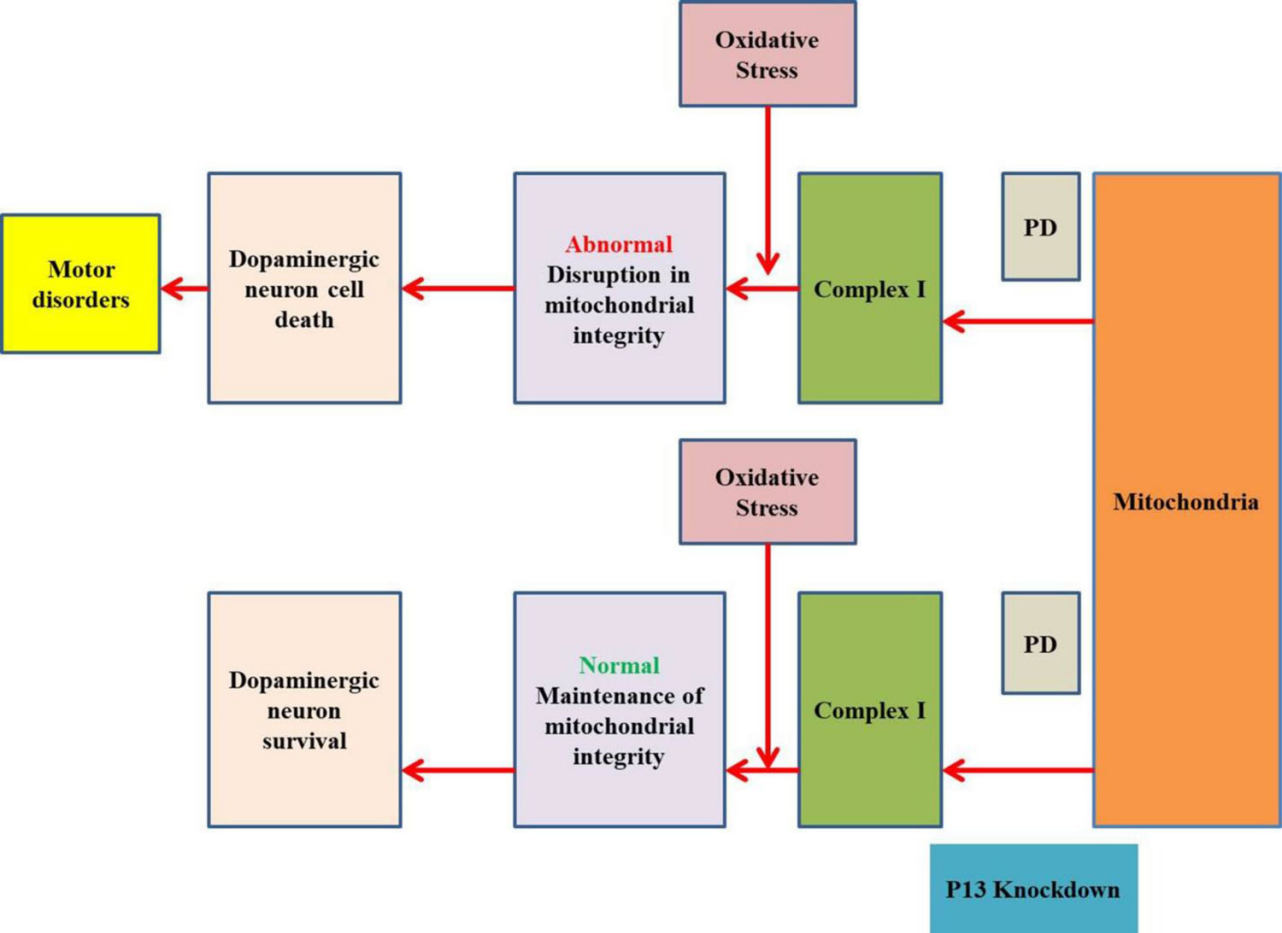
Down‐regulation of p 13 generates mitochondrial complex I assembly and inhibits cell death mediated by a mitochondrial abnormality in Parkinson's disease

Furthermore, Inoue et al. also discovered a subsequent reduction in the expression levels of p13 caused neuroprotective effects and rescued the genetic and toxin‐induced models of PD. Down‐regulation in the levels of p13 through utilizing shRNA prevented the neurotoxin‐induced disturbance of complex I assembly. On the other side, p13 overexpression enhanced the production of toxin‐induced phenotypes of PD models. Moreover, in further investigation, it is observed that the reduction of p13 in toxin‐induced models of PD taken place as a response to mitochondrial abnormality and cellular harm. However, a decrease in the level of p13 could secure mitochondria from the aggregation of toxins and hyper‐stabilize the Tne complex 1 (Figure 2).

Subsequently, Inoue et al. generated a knockout mouse that lacked expression of p13 by CRISPR/Cas9. Incredibly, these p13 knockout mice indicated no motor dysfunction or DAergic neuron destruction following therapy of MPTP. Furthermore, findings confirmed that in p13 knockout mice, complex I assemblage took place more consistently with the exposure of toxin. Therefore, mitochondrial activity has been preserved at a heightened level, which preserved normalcy neural cells (Inoue et al., 2018).

### 6.4 PK2 gene editing by clustered regularly interspaced short palindromic repeats

Latest studies have revealed a crucial function of PK2 in mitochondrial biogenesis. However, it is confirmed that the signaling of PK2 is improved in PD postmortem brains. Moreover, facing the neurotoxic stress leads to overexpression of PK2 that maintains mitochondrial biogenesis by enhancing the levels of BCL‐2, as well as, PGC‐1α and recovers loss in ATP synthesis mediated by neurotoxic agents (Gordon et al., 2016). In the MPTP‐induced gliosis model, PK2 overexpression reduced the reactive astrocyte mechanisms and enhanced the generation mechanism of neuroprotective A2 astrocyte phenotype (Neal et al., 2018). This counterbalancing effect can neutralize the neuroinflammation caused by reactive A1 astrocytes (Liddelow et al., 2017).

**FIGURE 3:**
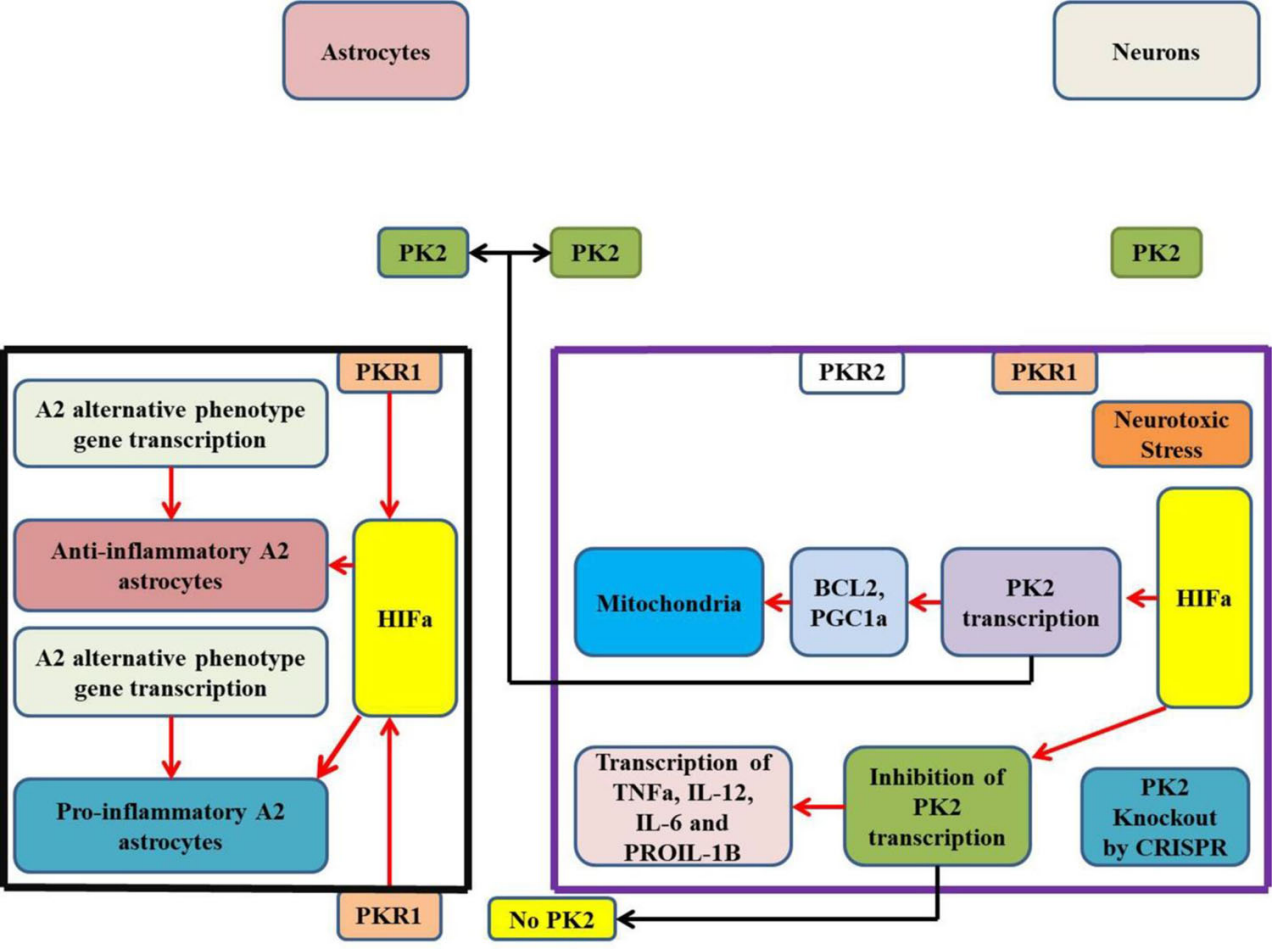
The knocked‐out prokineticin‐2 (PK2) regulated through clustered regularly interspaced short palindromic repeats/Cas9 shows the defensive function of PK2 in the antiapoptotic response to neurotoxic stress for either astrocytes or neurons. Neurotoxic stress can stimulate various transcription factors like hypoxia‐inducible factor 1‐alpha (HIF1α). In addition, this transcription factor links to the PK2 gene promoter and modulates this protein with expression. PK2 promotes mitochondrial biogenesis and modulates BCL‐2 and PGC‐1α, lead to cell ability of survival. Furthermore, PK2 in astrocytes modulates gene expression linked with anti‐inflammatory alternative activation (A2) phenotype. The ablation of the PK2 gene through CRISPR‐Cas9 generates unintended effects and enhances the susceptibility of the cell to neurotoxic stress

Furthermore, to validate the neuroprotective impact of PK2, CRISPR/Cas9 technique was applied to inhibit this gene with its receptor. Findings of these in vitro analyses showed that PK2 knockout improved the neural vulnerability to cell death induced by neurotoxic agents (Gordon et al., 2016). Moreover, knockout of PKR1 mediated by CRISPR/Cas9, an end to the effects of PK2 on the stimulation of alternate A2 astrocytes (Neal et al., 2018). Together with these findings reflect light on the relationship of neuroinflammation with mitochondrial dysfunction and show the use of CRISPR/Cas9 in specifying these processes (Figure 3).

### 6.5 PKCδ gene editing by clustered regularly interspaced short palindromic repeats

Prolonged therapy of endosulfan induces the apoptotic cascade, including the release of cytochrome‐c, stimulation of caspases, fragmentation of DNA, and proteolytic stimulation of PKCδ. In N27 DAergic cells, PKCδ was knock‐downed by the use of CRISPR/Cas9 to indicate the function of PKCδ in endosulfan‐mediated cell death. These balanced knock‐down cells showed a decrease in levels of caspase‐3 induced by endosulfan, which indicates that PKCδ plays a regulatory role in endosulfan‐induced apoptosis. Further analysis by utilizing autophagy and caspase‐2 or caspase‐3 inhibitors have shown that autophagy is the upstream mechanism compared to apoptosis and plays protective roles in application with endosulfan. Moreover, these findings suggest that autophagy dysfunctions play crucial roles in PD etiology; however, findings of the effective autophagy enhancer can be considered into concern as a possible PD therapy (Song et al., 2019).

## 7 PARKINSON'S DISEASE MODELING

Models of animals are feasible tools to study various neurological disorders, including PD. Since PD has heterogeneous etiology, different techniques have been applied to trigger the disorder in the models of an animal. These techniques include the use of neurotoxins and pharmaceutical products, which cause abnormalities related to midbrain DAergic signaling. Moreover, it includes the genetic manipulation approaches in the modeling of genetic forms of PD. Subsequently, 6‐hydroxydopamine and MPTP were widely applied to create pharmacologically based PD models (Dawson et al., 2018). However, applied models were proper in symptomatic treatments of motor manifestations in PD, while they were not acceptable in treatment strategies (Athauda & Foltynie, 2016). Furthermore, efforts have been made toward engineering genetic‐based PD models. Expectedly, CRISPR has emerged as a genuine technology in the field of modeling genetic animals with regard to the tissues of PD.

### 7.1 Modeling of in vivo Parkinson's disease by the clustered regularly interspaced short palindromic repeats/Cas9

In past decades, researchers have been using models of a transgenic mouse expressing wild form or mutant α‐synuclein and to investigate the PD on the basis of molecular pathophysiology. Up to date, some transgenic models have been created with the possibility to reflect the specific and cardinal pathological traits of the human PD, such as the actual immunopositive pathology of α‐synuclein or gradual destruction of DAergic neuron in SN (Blesa & Przedborski, 2014; Chesselet & Richter, 2011). Since PD pathogenesis takes longer than a mouse‐lifespan, the generation of the longer life span and PD models of a large animal is considered acceptable (Zhu et al., 2018).

Specifically, minipigs and pigs may be regarded as an appropriate model of large animals for human disorders (Yao et al., 2016). There are significant benefits to the minipigs that include a long decade of lifespan, size of the body comparable to humans, as compared to the rodents. Minipigs and pigs have short periods of gestation (such as four months). Also, the number of offspring is large of one pregnancy (over ten piglets), in addition to the existing onset of adolescence (five to six months). Furthermore, the similarities in the background of neuroanatomy and neurophysiology of minipigs with humans make them a functional tool in relation to neurodegenerative diseases (Dolezalova et al., 2014).

In this background, CRISPR/Cas9 was applied to create minipigs of Guangxi Bama harboring PD inducing SCNA mutations, such as, G51D, H50Q, and E46 K. Somatic cells gene editing was followed by the somatic cell nuclear transfer. Furthermore, the CRISPR/Cas9 and somatic cell nuclear transfer combination resulted in minipigs indicated PD‐particular pathological alterations, such as α‐synuclein immunopositive pathology as well as DAergic neuron loss in SN (Zhu et al., 2018).

As stated earlier, inherited mutations recessively in the genes PINK1, DJ‐1, and Parkin is closely related to familial types of early‐onset PD (Bonifati et al., 2002). The lack of an appropriate model is a crucial barrier to the generation of effective treatment strategies for humans PD. However, this lack of proper models has been unique to the PD of humans. Currently, a triple knockout has been created to upgrade our knowledge about the pathophysiology of PD in the model of pig and enhance symptomatic regulation of Parkin, PINK1, and DJ‐1 in them. Furthermore, in this attempt, the CRISPR/Cas9 technique was applied to alter the genes biallelically. Moreover, in this analysis, dual sgRNAs were used for targeting a particular gene, which significantly improved CRISPR/Cas9‐mediated genome editing (Wang et al., 2016).

### 7.2 Modeling of in vitro Parkinson's disease by clustered regularly interspaced short palindromic repeats/Cas9

IPSC technology delivers a potential possibility to generate a diverse range of modeling for PD disorder. Also, these models promote the study of pathological impacts of LRRK2 (LRRK2‐G2019S) mutation, such as raising the sensitivity of stress and decreasing neurite complexity (Nguyen et al., 2011; Sánchez‐Danés et al., 2012). Since the genetic context is a crucial factor in comparing patients with the control group, and isogenic cell lines with similar genetic contexts vary only in inserted mutations. Numerous LRRK2‐G2019S isogenic cell lines were created, including technologies such as Cre/LoxP and zinc finger systems (Reinhardt et al., 2013).

The main limitation in the Cre/LoxP system is the deletion of the chromatin, such as, a 34‐bp‐long LoxP, typically in the intron juxtaposed towards the site where mutations are corrected or introduced. It has been confirmed that deleting this LoxP site in an intron exerts impacts on the targeted allele expression (Zou et al., 2011). In mutations such as heterozygous, random incorporation of the LoxP site in unspecified regulating factors in an intron can also affect other gene regulation (Meier et al., 2010). These limitations have motivated the research society to create footprint‐free isogenic cell lines.

The efficiency and simplicity of CRISPR/Cas9 were compared to TALENs, and zinc‐finger nucleases highlight the supremacy of this technique for the editing of genes (Gaj et al., 2013). In the latest analyses, the CRISPR/Cas9 technique was used effectively for introducing mutations by the insertion of the reporter into the LRRK2 gene (Qing et al., 2017). In addition to the CRISPR technique, piggyback transposon was also employed to fully detach the chosen cassette to confirm the creation of a footprint‐free isogenic cell line (Ye et al., 2014). This isogenic cell line of LRRK2‐G2019S describes PD‐particular phenotypes, including neurite complexity, and generates a new way of understanding the function of S129P‐αS (Qing et al., 2017).

In some other in vitro models of PD, isogenic human embryonic stem cell lines, even with the RAB39B‐deletion, have been created. These models of cells will open a new way to understand PD pathogenesis properly. Moreover, they can also improve our knowledge of the RAB39B role. Moreover, in this analysis, the parental male human embryonic stem cell lines were transfected through CRISPR guidance, targeting both exon‐1 and exon‐2 of the RAB39B. However, these human embryonic stem cell lines indicate common morphological traits like their unaltered familial lines, such as tightly and small clustered cells, higher nucleus to cytoplasm proportion, and prominent nucleoli. By applying Yamanaka's factors, RAB39B knocked out human embryonic stem cell lines transformed to iPSC, which was validated by flow cytometry. Furthermore, this iPSC cell line may differentiate into the models of neurons to research the pathogenic processes of PD (Gao et al., 2018).

## 8 LIMITATIONS OF CLUSTERED REGULARLY INTERSPACED SHORT PALINDROMIC REPEATS FOR INDUCED PLURIPOTENT STEM CELLS GENOME EDITING

Cell reprogramming problems have emerged employing these strategies for therapy techniques such as the investigation of genomic dysfunctions during long‐term culture. (Hussein et al., 2011). Results of cytogenetic and molecular analyses showed the emergence of unintended chromosome aberrations, silent point mutations, polyploidies, and aneuploidies (Dekel‐Naftali et al., 2012).

The products of mutant genes can induce the instability of nuclear and mitochondrial genomes (Sanders et al., 2014). This is a significant phenomenon, and for genome editing, it must be taken into an investigation. Moreover, the drawback of iPSC genome editing is the susceptibility of the cells to DNA degradations, which leads to homological insertion with lower frequency. Furthermore, to investigate this, experiments conducted a cytogenetic study of nerve cells carrying modified G2019S mutation in the gene such as LRRK2 mediated by CRISPR/Cas9. Results of this study have confirmed a mosaic form of tetraploidy 92 XXYY/46, XY in 24–43% of cells derived even from different clones in the neuronal precursors differentiated from the iPSC. Moreover, translocations with single cases and breaks of the chromosome were confirmed. Furthermore, these findings suggest the importance of the latest techniques maintaining genome balance in CRISPR/Cas9‐modified cell cultures. Also, this finding can indicate whether each gene‐edited culture must be carefully determined for the presence of possible mutations before autological transplantation (Vetchinova et al., 2018). It is hypothesized that the genome aberrations independently emerge with the application of transcription factors non‐integration/integration techniques suggested for reprogramming (Gore et al., 2011).

The off‐target‐frequency effect is a serious challenge for genome editing by CRISPR‐Cas9. However, Cas9 has been expressed in the plasmid stage that could support the mismatches of single and double‐base, which leads to unintended mutagenesis (Fu et al., 2013). Creating accurate web‐based strategies for modeling specific gRNA and application of recombinant Cas9 protein, which destroys directly after exerting its intentional gene‐editing effect, can also decrease the off‐target effects. Furthermore, an effective delivery strategy for approaching the CNS framework is another limitation of CRISPR use. Lentivirus, AAV, Electroporation, microinjection, and liposomal or nanoparticle are typical delivery techniques, and some of these are functional for the CNS delivery of the CRISPR/Cas9‐based treatment (Arruda et al., 2017). Moreover, to overcome this restriction, a neuron‐preferring chimeric AAV‐based CRISPR‐Cas9 technique has been generated for triggering brain‐particular gene deletion (Murlidharan et al., 2016).

## 9 FUTURE TRENDS OF CLUSTERED REGULARLY INTERSPACED SHORT PALINDROMIC REPEATS FOR INDUCED PLURIPOTENT STEM CELLS GENOME EDITING

CRISPR enhances biomedical research and treatment strategy for PD as an emerging genome‐editing technique (Solberg & Krauss, 2013). This system provides the possibilities for all forms of genome alterations such as deletion of the sequences of long nucleotide or homologous recombination, in/del point mutations, and transcription manipulation of particular genetic elements. CRISPR/Cas9 is the most powerful technique for the technology of genome editing in human disease modeling in vivo and in vitro due to its functional nature and resistance to epigenetic changes (Soldner et al., 2016). Recently, CRISPR/Cas9 mediated genome editing of somatic or stem cells has become increasingly relevant. Also, in the same background, iPSCs have become effective models in understanding a specific pathology of the patient. However, these specified cell lines can be applied as a patient in the tube to create various clinical medications (Vasil'eva et al., 2015). Target alterations mediated by CRISPR/Cas9 in the iPSC genome deliver a vital cell line for the identification of genes associated with pathogenesis at different stages of the disorder in either in vitro or in vivo models.

## 10 CONCLUSION

Neurodegenerative disorders, particularly Parkinson's disease, are disabling for the patient and their family members and mostly impose a rising financial load on the health care system. Also, the gap between the research and the treatment has stayed completely open. Subsequently, to fill this gap, systems of CRISPR‐based editing of the genome in the neurons of human has been established. These systems promote the detection of cellular factors in regulating the susceptibility of the cellular mechanisms underlying PD neurodegeneration. The CRISPR/Cas9, iPSCs, and genome‐wide association studies technologies show the crucial factors behind the pathogenesis of PD. This can lead to the direction of introducing feasible clinical targets. We suggest using some other forms of CRISPR system such as Cas12a (Cpf1) to cope with the limits of CRISPR/Cas9, also with fewer unintended results. The benefits of Cas12a such as the potential to select T‐rich motifs even without the trans‐activating crRNA, the induction of staggered DSB, the ability for processing of RNA, and the potential for the activity of DNA nuclease. This programmable nuclease can be genuine guidance for us in exploring various clinical interventions for PD and several other neurodegenerative diseases.

## AUTHOR CONTRIBUTION

All the authors have accepted responsibility for the entire content of this submitted manuscript and approved submission.

## CONFLICT OF INTEREST

The authors declare that they have no competing interests.

### PEER REVIEW

The peer review history for this article is available at https://publons.com/publon/10.1002/brb3.2280.
